# Editorial: Antimicrobial resistance in food-producing environments: a One Health approach

**DOI:** 10.3389/frabi.2024.1436987

**Published:** 2024-06-19

**Authors:** Getahun E. Agga, Kebede Amenu

**Affiliations:** ^1^ Food Animal Environmental Systems Research Unit, Agricultural Research Service, United States Department of Agriculture, Bowling Green, KY, United States; ^2^ College of Veterinary Medicine and Agriculture, Addis Ababa University, Bishoftu, Ethiopia; ^3^ Animal and Human Health Program, International Livestock Research Institute (ILRI), Addis Ababa, Ethiopia

**Keywords:** antimicrobial resistance, One Health, antimicrobial use, antimicrobial stewardship, animal manure, probiotics, extended-spectrum β-lactamases, flies

## Introduction

The One Health High-Level Expert panel comprised of the United Nations Food and Agriculture Organization (FAO), the United Nations Environment Program (UNEP), the World Health Organization (WHO), and the World Organization for Animal Health (WOAH; founded as OIE) defines One Health as “an integrated, unifying approach that aims to sustainably balance and optimize the health of people, animals and ecosystems. It recognizes that the health of humans, domestic and wild animals, plants, and the wider environment (including ecosystems) are closely linked and interdependent. The approach mobilizes multiple sectors, disciplines and communities at varying levels of society to work together to foster well-being and tackle threats to health and ecosystems, while addressing the collective need for clean water, energy and air, safe and nutritious food, taking action on climate change, and contributing to sustainable development.” (Mettenleiter et al., 2023). There is nothing more fitting than antimicrobial resistance (AMR) to the principles of One Health, which provides a framework for an interdisciplinary approach to dealing with this global challenge (FAO, 2016; Robinson et al., 2016; Lancet, 2023).

Globally, bacterial AMR has been associated with an estimated 4.95 million human deaths in 2019, including 1.27 million deaths directly attributable to bacterial AMR (Murray et al., 2022). In the United States, bacterial AMR causes more than 2.9 million infections each year, with an estimated 35,000 deaths (CDC, 2019). Such estimates on the burden of antimicrobial-resistant pathogens to animal health do not exist (Robinson et al., 2016). Nevertheless, a recent ecological study (Allel et al., 2023) reported a 24.8% AMR mean prevalence in bacteria associated with food-producing animals. The authors also reported significant associations between animal antimicrobial consumption and AMR in bacteria associated with food-producing animals, and between human antimicrobial consumption and AMR in human pathogens. They also found bidirectional associations between veterinary antimicrobial sales and AMR in human pathogens, and between human antimicrobial sales and AMR in animals. The study highlighted that AMR in food-producing animals is associated with the quantity of antimicrobials sold for use in animals and in humans. Seven articles published under this theme discuss (1) baseline occurrence of AMR under cow-calf production; (2) estimation of national antimicrobial use in food-producing animals; (3) standardization of variables and parameters used to quantify on-farm antimicrobial use (AMU); (4) animal manure storages such as lagoons that serve as a reservoir for antimicrobial resistance genes (ARGs); (5) dissemination of AMR through flies; (6) probiotics as a source of AMR determinants; and (7) potential interventions to reduce the burden of AMR.

## AMR in beef cow-calf production system

Beef cow-calf production is an important and initial segment in commercial beef production system. First, it provides calves that are finished in feedlots for beef production; second, when culled from operation, beef cows are processed mainly for ground beef production. Compared to feedlot and dairy cattle production settings, antimicrobials are used infrequently. Understanding the epidemiology of AMR under the low antibiotic selection pressure that exists in cow-calf production can serve as a baseline for AMR risk that can arise when cows and calves enter the beef supply chain. Previous studies, including by the lead author of the paper by Agga et al., revealed that Gram-negative clinically important antimicrobial-resistant bacteria and associated genes can be found in cow-calf population (Agga et al., 2016a, 2019; Agga et al., 2022b). The study by Agga et al., conducted in a cow-calf operation, targeted enterococci as the indicator organism for Gram-positive bacteria and reported that while tetracycline-resistant enterococci were abundant, macrolide resistance occurred at low abundance. Furthermore, the two species, *E. faecalis* and *E. faecium*, most implicated in nosocomial infections, were widely detected in the cow-calf operation. The authors also pointed out that the use of wheat as a cover crop may have additional value in mitigating AMR in livestock raised under a grazing system.

## Standardized methods for the quantification of AMU in food animals

Previous studies using randomized field trials indicated that AMU in feedlot cattle for approved indications increases AMR in bacteria and specific ARGs (Agga et al., 2016b, 2023). Collecting on-farm AMU data is challenging and only a few countries have on-farm AMU monitoring systems. Most other developed countries including the United States use national sales data. Magiri et al. used import data to quantify AMU in Fiji. Since many developing countries rely on imported antimicrobials from developed countries, this method may be a practical approach in quantifying national antimicrobial purchases intended for use in animals. The use of different measurements and analytical approaches without standardization hinders direct evaluation of intervention efforts to reduce AMR. To overcome this challenge, Lu et al. compared different metrics and developed standardized methods of quantifying AMU, primarily obtained from sales data for use in food animals. The authors identified various AMU indicators, generally grouped as count-based, mass-based, and dose-based, that have been used to quantify AMU in animals; developed them into standardized approaches; and evaluated them for accuracy without compromising privacy for on-farm use and for their applicability to antimicrobial stewardship programs. The authors also identified limitations of the AMU quantification approaches such as lack of strong causal evidence between AMU and AMR among animal pathogens and commensals, and failure to include important information such as disease conditions, routes of administration, and antimicrobial resistance information into the AMU metrics.

## Environmental dissemination of AMR from animal farms

Animal manure is applied to fields following storage, and incentives are necessary for farmers to use existing manure-management technologies, which also have other added value such as biofuel production from anaerobic digestion and lagoon systems, and compost from animal manure composting, which can be used as organic fertilizer (Agga et al., 2022a). Animal manure removed from production facilities is stored in ponds, storage pits, or stockpiled between land applications. These storage units may act as reservoirs for AMR. Neher et al. compared the distribution of ARGs in manure storage pits across swine farms in Iowa. The study revealed that tetracycline and macrolide resistance genes were detected in all swine farms (n = 48) studied; their concentrations significantly varied among the farms, and by integrator type, with no significant effect of production type.

Caderhoussin et al. examined the role of flies in disseminating extended-spectrum β-lactamase (ESBL)-producing *Enterobacteriaceae* on cattle farms. By applying advanced molecular techniques, the origin and maintenance of ESBL-producing *Enterobacteriaceae* were investigated in a farm that raised food-producing animals with no history of third-generation cephalosporin uses. The study found a similarity between plasmids and genes of ESBL-producing *E. coli* strains isolated from flies and cattle. The findings from the study suggest that flies can act as effective mechanical vectors in transferring ARGs across environments and to multiple hosts. The study uncovered the complexity of factors responsible for the transfer and maintenance of ARGs. To address the complexity of AMR spread, a comprehensive One Health approach that integrates human, animal, and environmental health aspects is needed.

## Probiotics as a source of antibiotic-resistant bacteria and genes

Alternatives to antibiotics, including probiotics, are an important area of research to address the loss of effective treatments of infections caused by antimicrobial-resistant bacteria. Probiotics are direct-fed microbials that are added to animal feed to improve production efficiency and animal health (Cameron and McAllister, 2019). However, probiotics such as lactobacilli may be resistant to antibiotics, and spread AMR elements to commensal or pathogenic bacteria. Nøhr-Meldgaard et al. characterized diverse strains of bacteria in the *Lactobacillaceae* family for AMR, genotypically using whole genome sequence data for the presence of ARGs, and phenotypically by minimum inhibitory concentration (MIC) method using epidemiological cutoffs (ECOFF). The authors used phylogenetic relatedness, rather than the traditional fermentation-based method, to propose a new ECOFF for the family of bacteria. Almost in all strains, acquired ARGs were not detected, thus fulfilling one of the theoretical requirements for a probiotic.

## AMR interventions

Jacobsen et al. conducted a scoping review to summarize the literature reporting AMR interventions in animals. The study provided a comprehensive overview of various interventions and tools aimed at reducing AMU and AMR in the animal health sector by classifying the interventions into different major categories: (1) change in AMU practices, (2) change in the uptake of antimicrobial stewardship (AMS), (3) change in the development of AMR, (4) change in the knowledge of AMR and change in the knowledge of appropriate AMU and AMS practices, (5) change in attitudes and perceptions concerning AMU, AMR, and AMS, and (6) surveillance strategies. The review indicated that only one-fifth of the reviewed papers targeted developing countries. The review revealed that objective means of evaluating the interventions are not common, but self-reported subjective responses are. Specifically, financial aspects are not considered when interventions are evaluated. The authors assert that a full understanding of the interlinked global efforts toward evaluating interventions requires proportional coverage in developed and developing countries by using objective metrics and targeting financial aspects.

## Conclusions

The theme One Health approach to AMR in food-producing animals attracted papers that evaluated the epidemiology and ecology of AMR including interventions taken to reduce it. The papers ranged from the baseline occurrence of AMR under low antibiotic selection pressure, AMR dissemination pathways such as animal manure, probiotics, and flies, AMU quantification and standardization, to the interventions taken to reduce AMR. The papers presented critical information needed to optimize AMU in food-producing animals and the roles of other factors in disseminating AMR.

## Author contributions

GA: Writing – original draft, Writing – review & editing. KA: Writing – original draft, Writing – review & editing.
